# Racial/Ethnic Disparities in Health and Life Insurance Denial Due to Cancer among Cancer Survivors

**DOI:** 10.3390/ijerph19042166

**Published:** 2022-02-15

**Authors:** Adrienne B. Lent, Carlos O. Garrido, Emily H. Baird, Ruta Viela, Robin B. Harris

**Affiliations:** 1 University of Arizona Cancer Center, University of Arizona, 1515 N Campbell Ave, Tucson, AZ 85724, USA; rbharris@email.arizona.edu; 2 Division of Cancer Control and Population Sciences, National Cancer Institute, 9609 Medical Center Drive, 3E638, Bethesda, MD 20892, USA; carlos.garrido@nih.gov; 3 Kinesiology and Public Health, California Polytechnic State University, San Luis Obispo, 1 Grand Avenue, San Luis Obispo, CA 93407, USA; ehbaird@calpoly.edu (E.H.B.); rviela@calpoly.edu (R.V.)

**Keywords:** race and ethnicity, health insurance, life insurance, Affordable Care Act, cancer survivors, cancer health disparities

## Abstract

This study examined racial/ethnic differences in health/life insurance denial due to cancer among cancer survivors after the passage of the Affordable Care Act (ACA). Behavioral Risk Factor Surveillance System data were obtained from 2012–2020. The dependent variable asked: “Were you ever denied health insurance or life insurance coverage because of your cancer?” Cancer survivors were included if they were diagnosed with cancer after the Affordable Care Act (*N* = 14,815). Unadjusted and adjusted logistic regressions for age, sex, income, and employment provided odds ratios of insurance denial due to cancer across racial/ethnic groups: Non-Hispanic White, Black, and Other/mixed race; and Hispanic. Statistically significant differences (*p* < 0.05) were found between those who were denied or not denied insurance across sex, age, race/ethnicity, income, and employment. Adjusted regressions found significantly higher odds ratios of insurance denial for Blacks (OR: 3.00, 95% CI: 1.77, 5.08), Other/mixed race (OR: 2.16, 95% CI: 1.16, 4.02), and Hispanics (OR: 2.13, 95% CI: 1.02, 4.42) compared to Whites. Differences were observed across sex, income, and employment. Cancer survivors report racial/ethnic disparities in health and life insurance denial due to their cancer despite policy changes. This may be harmful for those who are already financially vulnerable due to their cancer diagnosis and exacerbate racial/ethnic cancer disparities.

## 1. Introduction

Cancer is the second leading cause of death in the United States and over 16.9 million Americans living today have a history of cancer [1]. Cancer disproportionately affects underrepresented racial/ethnic groups. For example, Black/African Americans have the highest death rates for most cancer types while Hispanic and Black women have high rates of cervical cancer, a cancer that is preventable through screening [2,3].

Health insurance plays a significant role in cancer prevention and survival [4]. The Affordable Care Act (ACA) focused on decreasing the number of people without health insurance and on reducing racial/ethnic health disparities [5]. The ACA made health insurance available to millions of Americans through several provisions, including the expansion of Medicaid eligibility for individuals with low incomes, establishment of health insurance marketplaces with subsidies to those who qualify, extension of coverage for dependents to age 26, and elimination of insurance coverage restrictions for individuals with pre-existing conditions [6]. Since 2010, insurers are legally prohibited from denying health insurance to consumers over pre-existing conditions at the federal level. Gains in health insurance coverage from the ACA have been largest for Americans under the age of 65 who are not eligible for Medicare. Additionally, gains in health insurance coverage due to the ACA have been larger for Non-Hispanic Blacks and Hispanics compared to Whites [7]. Despite reaching an all-time low in the number of people without health insurance, people from underrepresented racial/ethnic groups continue to have the highest uninsured rates [8]. This is particularly concerning for individuals with a cancer diagnosis since uninsured cancer patients are twice as likely to experience financial toxicity and worse quality of life compared to insured cancer patients [9]. 

Life insurance provides compensation after the death of the insured individual and is important for the financial health and generational wealth of beneficiaries. This is especially true for those with low incomes who are more likely to belong to underrepresented racial/ethnic groups [10]. Pre-existing conditions may make obtaining life insurance more difficult and expensive, although not impossible [11,12,13]. Existing literature has documented historical discrimination in life insurance denial among people of color [14]. Black Americans have been either denied life insurance based on their race or paid for the same life insurance policies as White Americans, but the policies were worth significantly less despite identical premiums [14,15]. After the passage of the 1964 Civil Rights Act, insurance companies are no longer allowed to charge race-based insurance premiums [14]. 

While previous literature has focused on changes in the uninsured rate across groups since the ACA and denial of health insurance claims for treatments, studies have not examined health insurance denial experiences post-ACA [8]. Additionally, research has not examined whether underrepresented groups continue to experience higher rates of life insurance denial compared to nonunderrepresented groups. One study published in 2020 found that people who identify as transgender or nonbinary experience health insurance coverage denials for health services, and such denials differ by type of insurance [16]. Another study found that youth who identify as transgender experience high rates of insurance coverage denials for hormone therapy [17]. A 2020 study found that rates of having life insurance are about the same for Black and White people. However, Black people have one-third of the coverage despite having similar incomes [18]. Still unknown is whether racial/ethnic differences persist in the denial of health or life insurance coverage over pre-existing conditions. 

This study aims to fill these gaps in knowledge by analyzing annual survey data on health or life insurance denial due to a cancer diagnosis collected by the Behavioral Risk Factor Surveillance System (BRFSS) between 2012 and 2020. We hypothesized that cancer survivors from underrepresented racial/ethnic groups would report higher rates of insurance denial due to their cancer diagnosis compared to non-Hispanic whites. Our primary objective was to examine the association between race/ethnicity and health and life insurance denial due to a cancer diagnosis among cancer survivors after the ACA. 

## 2. Materials and Methods

The Behavioral Risk Factor Surveillance System (BRFSS) is a telephone-based survey of noninstitutionalized adults conducted by individual U.S. states annually that uses random digit dialing and multistage cluster sampling. Surveys are administered over both landline and cell phones with cell phone collection starting in 2008 [19]. BRFSS is considered the premier system of health-related telephone surveys in the U.S., which collects a nationally and state representative sample [19,20].

Surveys collect data on health risks, behaviors, and outcomes across a variety of conditions. A cancer survivorship module is included in both common and optional modules for states in varying years. 

Publicly available BRFSS data were obtained from survey years 2012, 2014, 2016, 2017, 2018, 2019 and 2020 and included 35 states, Guam, and the Virgin Islands. Among the 53,846 cancer survivors with available data for the outcome variable, participants were included in the study if they were diagnosed with cancer after the rollout of the Affordable Care Act (ACA) in 2010. Due to case definition, 30,500 individuals were excluded due to having a cancer diagnosis before the ACA and 8531 were excluded due to missing data, resulting in a final sample size of *n* = 14,815. Determination of a cancer diagnosis before or after the 2010 ACA was done by calculating the difference between their current age and age of cancer diagnosis for each survey year. 

The dependent variable consisted of responses to the composite BRFSS question, “Were you ever denied health insurance or life insurance coverage because of your cancer?” The responses were coded as a binary variable (yes or no). 

The main independent variable was race/ethnicity. The BRFSS survey categorized race/ethnicity as non-Hispanic White only, non-Hispanic Black only, non-Hispanic American Indian or Alaskan Native only, non-Hispanic Asian only, non-Hispanic Native Hawaiian or other Pacific Islander only, non-Hispanic Other race only, non-Hispanic multiracial, and Hispanic. Due to small sample sizes across some race/ethnicity groups, this variable was coded as non-Hispanic White only, non-Hispanic Black only, non-Hispanic Other, and Hispanic. Covariates included sex (male or female), age (18–39, 40–59, and 60+), income(<$25,000, ≥$25,000 to <$50,000, ≥$50,000 to <$75,000, and ≥$75,000 or more), education (finished high school or less, attended college/technical school, and graduated from college/technical school), employment status (employed or not employed), marital status (married or not married), and number of cancer diagnoses (one or two or more). 

Analyses were conducted using Stata Version 17 (StataCorp LP, College Station, TX, USA). BRFSS analysis requires weighting to adjust for noncoverage and nonresponse among the sample of respondents to equal population estimates for the geographic region and nationally. We utilized the CDC weights provided in the datasets. Variables were weighted using the BRFSS weighting factor (_LLCPWT) specific for each state and survey year to adjust for demographic differences between individuals included in the sample and those in the population they represent [21]. BRFSS weights for the following eight margins: gender by age group, race/ethnicity, education, marital status, tenure, gender by race/ethnicity, age group by race/ethnicity, and phone ownership [22]. 

Weighted percentages summarized data across demographic factors. Chi-square tests were used to evaluate comparisons between those denied and those not denied insurance due to cancer. Unadjusted and adjusted logistic regression was used to calculate odds ratios and 95% confidence intervals to examine the association between race/ethnicity and denial of insurance. Adjusted models included age, sex, income, employment status, and survey year. Models were adjusted for age, sex, income, and employment since these variables met the definition of a confounder. Survey year was included as a covariate to account for fluctuations in data across waves. Interactions were evaluated for income sex, age, and employment status. The potential for interactions between race and sex, income, and employment were examined using separate models that included interaction terms. Stratified results are presented with Wald statistic.

## 3. Results

### 3.1. Participant Characteristics

Table 1 shows weighted percentages for respondent demographics among cancer survivors diagnosed with cancer after the ACA for the overall sample and those who reported that they were denied or not denied insurance coverage due to cancer. Overall, 4.58% of respondents reported being denied health or life insurance due to their cancer. The overall sample tended to be male (50.19%), over the age of 60 (63.09%), White (88.88%), have an annual income >$75,000 (39.00%), high school educated or less (35.54%), married (65.18%), employed (42.06%), and have one cancer diagnosis (88.92%). 

Being denied health insurance varied by sex, age, and race (Table 1). Respondents were more likely to be denied if they were female (5.45%) compared to males (3.71%), ages 18–39 years (8.86%) and ages 40–59 years (6.87%) compared to over 60 years (3.02%), and non-White ethnicity (11.00% of Blacks, 10.00% of Hispanics, and 9.30% of Other) compared to Whites (3.85%). Income also affected whether insurance was denied with those earning less than $25,000 per year (6.22%) being more likely to be denied than those earning more (3.43% for $25,000–50,000, 4.37% for $50,000–75,000, and 4.58% for $75,000+). Employment status also affected whether insurance was denied with the employed (5.99%) and not employed (5.80%) being more likely to be denied insurance than those who were retired (2.70%). 

### 3.2. Main Results

Table 2 shows adjusted and unadjusted odds ratios of the associations between race/ethnicity and denial of insurance coverage due to cancer. In comparison to the reference group of White respondents, non-Hispanic Black (adj. OR = 3.00, 95% CI: 1.77, 5.08), non-Hispanic other/mixed race (adj. OR = 2.16, 95% CI: 1.16, 4.02), and Hispanic (adj. OR = 2.13, 95% CI: 1.02, 4.42) respondents experienced significantly higher odds ratios of being denied health or life insurance coverage due to their cancer diagnosis. Making less than $25,000 per year (adj. OR = 1.53, 95% CI: 1.04, 2.26) and being retired (adj. OR = 0.62, 95% CI: 0.44, 0.85) were significantly associated with insurance denial. 

### 3.3. Stratified Results

Table 3 displays adjusted odds ratios for associations between race/ethnicity and insurance denial stratified by levels of sex, income, and employment status. Compared to non-Hispanic White males, the odds ratio of insurance denial among non-Hispanic White females was 1.51 (95% CI: 1.12, 2.04). Compared to non-Hispanic White males, the odds ratios of insurance denial among non-Hispanic Black males was 3.41 (95% CI: 1.56, 7.42), which rose to 4.13 (95% CI: 2.08, 8.24) among non-Hispanic Black females. Compared to non-Hispanic White males, the odds ratios of insurance denial were higher among non-Hispanic Other males (adj. OR = 3.90, 95% CI: 1.54, 9.85) and Hispanic males (adj. OR = 3.33, 95% CI: 1.11, 10.01). Compared to non-Hispanic Whites making ≥$75,000 per year, the odds ratio of insurance denial among non-Hispanic Whites making <$25,000 per year was 1.81 (95% CI: 1.22, 2.67). Compared to non-Hispanic Whites making ≥$75,000 per year, the odds ratios of insurance denial among non-Hispanic Blacks was highest for individuals making ≥$50,000 to <$75,000 (adj. OR = 6.81, 95% CI: 2.13, 21.73) but remained higher for those making ≥$25,000–<$50,000 (adj. OR = 3.35, 95% CI: 1.36, 8.26) and <$25,000 per year (adj. OR = 4.14, 95% CI: 1.55, 11.07). Compared to non-Hispanic Whites making ≥$75,000 per year, the odds ratio of insurance denial among non-Hispanic Others was 3.00 (95% CI: 1.09, 8.25) for those making ≥$75,000, which rose to 9.61 (95% CI: 3.21, 28.80) for those making $50,000–<$75,000 per year. Compared to non-Hispanic Whites making ≥$75,000 per year, the odds ratio of insurance denial among Hispanics making ≥$75,000 per year was 4.91 (95% CI: 1.68, 14.35). Compared to employed non-Hispanic Whites, the odds ratios of insurance denial among employed non-Hispanic Blacks was 3.66 (95% CI: 1.61, 8.31). Compared to employed non-Hispanic Whites, employed non-Hispanic Others had a higher odds ratio of insurance denial (adj. OR = 3.12, 95% CI = 1.41, 6.92). 

## 4. Discussion

The purpose of this study was to examine the association between race/ethnicity and health or life insurance denial due to a cancer diagnosis among those respondents diagnosed after the ACA. We found that even after adjusting for confounders, Non-Hispanic Blacks, non-Hispanic other/mixed race, and Hispanics were significantly more likely to be denied insurance due to their cancer diagnosis compared to non-Hispanic Whites. Blacks appeared to be most affected and experienced three times the risk of insurance denial due to their cancer compared to Whites. Other/mixed race and Hispanic participants were more than twice as likely to experience insurance denial due to cancer compared to Whites. Furthermore, denial differed across certain sex, income, and employment status categories. Compared to White males, Black, Other, and Hispanic males had a higher risk of insurance denial. While the risk of insurance denial was higher among Black males compared to White males, the risk of insurance denial was even higher for Black females. Compared to Whites making more than $75,000 per year, the risk of being denied insurance among Whites was higher for those in the lowest income level. Compared to Whites making more than $75,000 per year, the risk of insurance denial was higher among blacks across all income levels except for the highest earners making ≥$75,000. The risk of insurance denial was also higher among non-Hispanic Others and Hispanics in the highest income level making ≥$75,000 per year compared to non-Hispanic Whites with the same income. Compared to employed Whites, employed non-Hispanic Blacks, and non-Hispanic, Others had a higher risk of insurance denial. 

Discrimination may explain the observed associations with health or life insurance denial due to cancer. Blacks and Native Americans report significantly higher rates of perceived racial discrimination when accessing health care compared to Whites [23]. Hispanic/Latinx parents have reported discrimination as a barrier to enrolling their children in no-cost Medicaid despite them being eligible [24]. Racial/ethnic discrimination has also been observed within the home insurance industry. A 2006 study found that home insurance companies often use “linguistic profiling”, which occurs when the race/ethnicity of an individual is identified based on the sound of their voice. Home insurance agents are generally able to identify a customer’s race/ethnicity if contacted over the phone, which affects the services they are provided [25]. It is possible that this discrimination may also occur when obtaining health or life insurance. 

We found that the association between race/ethnicity and reported health or life insurance denial due to cancer differed across income. In general, the risk of reported insurance denial was higher for people of color in the higher income groups and among those who were employed. This is similar to previous research that found that Blacks with higher socioeconomic status report higher rates of perceived discrimination within the U.S. health care system compared to Blacks with low socioeconomic status [23]. This may be due to “racism-related vigilance” in which individuals mentally prepare for the possibility of experiencing race-based discrimination [26]. It is possible that a similar phenomenon may play an explanatory role in these findings and contribute to the higher reported insurance denial among underrepresented racial/ethnic groups compared to Whites. Successful enrollment and maintenance of insurance requires payment of premiums on time. Premium subsidies are generally available for Americans who make less than 400% of the federal poverty level, meaning that those in higher income categories may be ineligible for financial assistance [27]. Furthermore, Black and Hispanic individuals with cancer experience higher levels of financial toxicity related to their cancer compared to Whites [28]. This lack of health insurance premium assistance for those just above income thresholds combined with racial/ethnic disparities in financial toxicity may result in issues paying initial policy premiums, resulting in denial of coverage. 

We found that the association between race/ethnicity and reported health or life insurance denial due to cancer differed across sex. While White and Black women had a higher risk of insurance denial compared to White males, the risk of insurance denial was almost three times higher for Black women compared to White women. Women have historically faced discrimination when purchasing health insurance. This includes “gender rating”, which involves charging different premiums based on gender and results in significantly higher premiums for women [29]. While the ACA prohibits new individual and small group plans from gender rating, implementation and enforcement of ACA provisions remain a challenge [30]. It is possible that gender- and race-based discrimination persist, especially among women of color. 

We also found that the association between race/ethnicity and reported health or life insurance denial due to cancer differed across employment status. Employed individuals who identified as Black and Other were significantly more likely to be denied insurance compared to employed Whites. Individuals who identify as Black have the highest rate of fatal occupational injuries compared to other racial/ethnic groups [31]. It is possible that life insurance companies are denying coverage for individuals who work in high-risk occupations due to cost-related reasons. Additionally, unemployed individuals may be more likely to qualify for Medicaid health insurance, which has state-based eligibility criteria around income and disability status [32]. 

Enrolling in life and health insurance can be complex and difficult to navigate. This is especially true for racial and ethnic groups who may have lower health literacy. Prior research has found that Hispanics and Blacks have significantly lower levels of health insurance literacy compared to Whites [33,34]. Spanish speakers also experience lower levels of health literacy compared to English speakers [35]. Another study found that Black adults with cancer are significantly more likely to have lower cancer health literacy levels compared to White adults with cancer [36]. It is possible that insurance literacy plays an important role in the observed racial/ethnic disparities in reported health or life insurance denial due to a cancer diagnosis. Although we did control for education level, health literacy is not captured in the BRFSS data. While the BRSS survey is administered in both English and Spanish, language is not a variable provided in the dataset. 

Enrolling in life and health insurance requires providing a number of documents. Applicants who sign up for health insurance through the Marketplace via HealthCare.gov are required to provide supporting documentation and select a plan to complete their application [37]. Application assistance from insurance navigators has been shown to predict successful enrollment in Marketplace plans setup under the ACA [38]. One small study of just over 1000 individuals in the Southern U.S. found that Latinos but not Blacks were more likely to receive assistance from navigators when signing up for insurance compared to those who were White [39]. Interestingly, our study found that males across all race/ethnicity categories were more likely to be denied insurance due to their cancer. Although gender differences in receipt of insurance navigation assistance have not been observed in existing research, males have been less likely to seek health information in general compared to females [39,40]. It is possible that males are less likely to seek out and receive assistance from a navigator when enrolling in insurance through the Marketplace on a national level, although this is an area that should be further explored.

Life insurance companies generally financially incentivized to insure healthy individuals who will pay premiums over a long period of time [41]. While it is possible to get life insurance with cancer, having multiple chronic diseases increases the likelihood of a life insurance company denying an application for coverage. In the U.S., Blacks and Hispanics are more likely to have multiple chronic diseases at the same time compared to those who are White [42]. Among prostate cancer survivors, Blacks were more likely to be smokers and Blacks and Hispanics were more likely to have three to six chronic conditions compared to those who are White [43]. Since we are unable to tell whether health or life insurance is driving the observed racial/ethnic differences in our data and health insurance denial is no longer allowed after the ACA, it is possible that differences in the overall chronic disease burden experienced by people of color in the U.S. is resulting in an increased risk of life insurance denial, thus driving the observed racial/ethnic disparities. 

These findings have several limitations. First, this study used cross-sectional data and cannot establish causation. Second, the BRFSS question for the dependent variable asked participants if they had ever been denied health or life insurance due to their cancer diagnosis. Therefore, we are unable to determine whether the denial due to a pre-existing condition was specifically for health insurance, not allowed under the ACA, or life insurance, currently allowed in the U.S. However, it is important to note that insurance denial based on race is not allowed in the U.S. Third, BRFSS survey participants must have responded to a landline or cell phone survey conducted over the phone, which may have biased the sample. Fourth, all BRFSS variables are self-reported, and participants are asked to recall if they had ever been denied health or life insurance due to their cancer. Therefore, bias and under or overreporting is possible. Fifth, a Non-Hispanic Other category was created for the race/ethnicity categories, which included individuals who identified as American Indian or Alaskan Native, Asian, Native Hawaiian or other Pacific Islander, other race, and multiracial. Due to small sample sizes for those individual categories, we were unable to determine which individual group (s) drove the observed association with insurance denial. Last, this study controlled for individual income but did not account for household income, which could influence insurance denial. Despite these limitations, we were able to limit the sample to cancer survivors with a cancer diagnosis made after the ACA, thus eliminating the possibility of health insurance denial for a pre-existing condition when it was legal in the U.S. This analysis sample was also geographically diverse and included a large overall sample size.

## 5. Conclusions

Despite health insurance denial for pre-existing conditions being illegal under the ACA and policies that prohibit health and life insurance coverage based on race/ethnicity, racial/ethnic disparities in health and life insurance denial due to their cancer diagnosis were observed. This denial may be particularly harmful for people of color who are already financially vulnerable due to their cancer diagnosis and exacerbate racial/ethnic cancer disparities. Future studies should examine longitudinal data and include qualitative methods to better understand the experiences of people of color in obtaining health and life insurance to reduce racial/ethnic disparities.

## Figures and Tables

**Table 1:** Weighted percentages and unweighted counts of respondent demographics among cancer survivors by self-reported denial of health or life insurance, 2012–2020 BRFSS. (*n* = 14,815).

	Overall % (*n*)	Denied Insurance % (Row *n*)	Never Denied Insurance % (Row *n*)	*p*-Value *
Sex				0.007
Male	50.19 (7058)	3.71 (248)	96.29 (6810)	
Female	49.81 (7757)	5.45 (378)	94.55 (7379)	
Age				<0.001
18–39	6.82 (533)	8.86 (45)	91.14 (488)	
40–59	30.09 (3513)	6.87 (251)	93.13 (3262)	
60+	63.09 (10,769)	3.02 (330)	96.98 (10,439)	
Race/Ethnicity				<0.001
White, Non-Hispanic	88.88 (13,581)	3.85 (512)	96.15 (13,069)	
Black, Non-Hispanic	6.16 (552)	11.00 (52)	89.00 (500)	
Other, Non-Hispanic **	2.55 (453)	9.30 (42)	90.70 (411)	
Hispanic	2.42 (229)	10.00 (20)	90.00 (209)	
Income				0.032
<$25,000	19.57 (3054)	6.22 (162)	93.78 (2892)	
>$25,000–<$50,000	25.00 (3975)	3.43 (155)	96.57 (3820)	
>$50,000–<$75,000	16.43 (2581)	4.37 (87)	95.63 (2494)	
>$75,000	39.00 (5205)	4.58 (222)	95.42 (4983)	
Education				0.946
Finished high school or less	35.54 (4534)	4.57 (201)	95.43 (4333)	
Attended college/technical school	30.37 (4004)	4.72 (196)	95.28 (3808)	
Graduated from college/technical school	34.08 (6277)	4.47 (229)	95.53 (6048)	
Employment Status				<0.001
Employed	42.06 (5584)	5.99 (304)	94.01 (5280)	
Not employed	15.96 (1942)	5.80 (126)	94.20 (1816)	
Retired	41.98 (7289)	2.70 (196)	97.30 (7093)	
Marital Status				0.999
Married	65.18 (9058)	4.58 (384)	95.42 (8674)	
Not married	34.82 (5757)	4.58 (242)	95.42 (5515)	
Number of Cancer Diagnoses				0.302
One	88.92 (13,135)	4.47 (534)	95.53 (12,601)	
Two or more	11.08 (1680)	5.42 (92)	94.58 (1588)	
Total	100 (14,815)	4.58 (626)	95.42 (14,189)	

* chi-square *p*-value. ** Other includes (non-Hispanic): American Indian or Alaskan Native, Asian, Native Hawaiian or other Pacific Islander, other race, and multiracial.

**Table 2:** Unadjusted and adjusted odds ratios for the association between race/ethnicity and health or life insurance coverage denial due to cancer among cancer survivors according to the 2012–2020 BRFSS.

	Unadjusted OR (95% CI)	*p*-Value	Adjusted OR * (95% CI)	*p*-Value
Race/Ethnicity				
White, Non-Hispanic	1.0		1.0	
Black, Non-Hispanic	3.08 (1.79, 5.32)	<0.001	3.00 (1.77, 5.08)	<0.001
Other, Non-Hispanic **	2.56 (1.42, 4.60)	0.002	2.16 (1.16, 4.02)	0.015
Hispanic	2.77 (1.39, 5.51)	0.004	2.13 (1.02, 4.42)	0.043
Sex				
Male			1.0	
Female			1.33 (1.00, 1.77)	0.046
Income				
>$75,000			1.0	
>$50,000–<$75,000			1.11 (0.73, 1.70)	0.616
>$25,000–<$50,000			0.95 (0.67, 1.35)	0.779
<$25,000			1.53 (1.04, 2.26)	0.032
Employment				
Employed			1.0	
Not Employed			0.77 (0.53, 1.11)	0.161
Retired			0.62 (0.44, 0.85)	0.004

* Adjusted for variables in the table and age and survey year. ** Other includes (non-Hispanic): American Indian or Alaskan Native, Asian, Native Hawaiian or other Pacific Islander, other race, and multiracial.

**Table 3:** Adjusted odds ratios for the effect of race/ethnicity and health or life insurance coverage denial due to cancer stratified by levels of selected demographic factors.

	Adjusted OR (95% CI) *				
	White, Non-Hispanic	Black, Non-Hispanic	Other, Non-Hispanic **	Hispanic	*p*-Value ***
Sex					0.09
Male	1.0	**3.41 (1.56, 7.42)**	**3.90 (1.54, 9.85)**	**3.33 (1.11, 10.01)**	
Female	**1.51 (1.12, 2.04)**	**4.13 (2.08, 8.24)**	2.05 (0.92, 4.60)	2.32 (0.94, 5.75)	
Income					0.03
>$75,000	1.0	1.85 (0.71, 4.86)	**3.00 (1.09, 8.25)**	**4.91 (1.68, 14.35)**	
>$50,000–<$75,000	1.01 (0.65, 1.59)	**6.81 (2.13, 21.73)**	**9.61 (3.21, 28.80)**	0.31 (0.37, 2.58)	
>$25,000–<$50,000	0.94 (0.65, 1.37)	**3.35 (1.36, 8.26)**	2.06 (0.81, 5.22)	2.21 (0.64, 7.56)	
<$25,000	**1.81 (1.22, 2.67)**	**4.14 (1.55, 11.07)**	1.37 (0.53, 3.57)	1.77 (0.56, 5.57)	
Employment Status					0.27
Employed	1.0	**3.66 (1.61, 8.31)**	**3.12 (1.41, 6.92)**	2.61 (1.00, 6.83)	
Not Employed	0.88 (0.61, 1.23)	2.36 (0.93, 5.97)	0.82 (0.26, 2.60)	1.24 (0.40, 3.80)	
Retired	**0.68 (0.48, 0.97)**	1.54 (0.65, 3.68)	1.36 (0.39, 4.74)	1.21 (0.22, 6.58)	

* Adjusted for age, survey year, and other variables in the table. ** Other includes (non-Hispanic): American Indian or Alaskan Native, Asian, Native Hawaiian or other Pacific Islander, other race, and multiracial. *** Wald statistic *p*-value reported for interaction terms included in adjusted logistic regression models.

## Data Availability

The data can be downloaded from the CDC website: https://www.cdc.gov/brfss/annual_data/annual_data.htm (accessed on 24 November 2021).
